# IMPlementing IMProved Asthma self-management as RouTine (IMP^2^ART) in UK primary care: An internal pilot for a cluster randomised controlled trial

**DOI:** 10.1371/journal.pone.0336745

**Published:** 2026-03-20

**Authors:** Kirstie McClatchey, Daisy Bertram, Atena Barat, Brigitte Delaney, Vicky Hammersley, Barbara Korell, Viv Marsh, Megan Preston, Jessica Sheringham, Liz Steed, Steven Julious, David Price, Stephanie J. C. Taylor, Hilary Pinnock

**Affiliations:** 1 Asthma UK Centre for Applied Research, Usher Institute, The University of Edinburgh, Edinburgh, United Kingdom; 2 Directorate of Public Health, NHS Tayside, Dundee, United Kingdom; 3 Wolfson Institute of Population Health, Queen Mary University of London, London, United Kingdom; 4 SCHARR, The University of Sheffield, Sheffield, United Kingdom; 5 NHS Black Country Integrated Care Board, Wolverhampton, United Kingdom; 6 Optimum Patient Care, Oakington, United Kingdom; 7 hVIVO Services Ltd, London, United Kingdom; 8 University College London, London, United Kingdom; 9 Observational and Pragmatic Research Institute, Singapore, Singapore; 10 Centre of Academic Primary Care, Division of Applied Health Sciences, University of Aberdeen, Aberdeen, United Kingdom; Queensland University of Technology, AUSTRALIA

## Abstract

**Introduction:**

Supported self-management that includes a personalised asthma action plan and regular professional review, reduces unscheduled consultations, and improves asthma outcomes and quality of life. However, despite unequivocal inter/national guideline recommendations, supported self-management is poorly implemented in UK primary care. The IMPlementing IMProved Asthma self-management as RouTine (IMP^2^ART) implementation strategy (including facilitated provision of patient, professional, and organisational resources) has been developed to address this challenge and is being evaluated in a UK-wide cluster randomised controlled trial (cRCT). The internal pilot aimed to assess the trial recruitment processes, delivery of and general practice engagement with the implementation strategy to inform the progression criteria.

**Methods:**

Using mixed methods, we recruited 12 general practices and monitored trial processes and IMP^2^ART delivery through team logs and automated data (e.g., use of patient online resources; uptake of practice education modules). Qualitative interviews with general practice staff and IMP^2^ART facilitators explored feasibility and acceptability of the implementation strategy.

**Results:**

We randomised 12 general practices to the IMP^2^ART implementation strategy arm (n = 6) or usual asthma care (control, n = 6). One control practice withdrew post-randomisation following concerns about data sharing. Most components were delivered successfully to the implementation group practices so that we met our progression criteria. In all six practices, the facilitated workshop was arranged within 12 weeks of randomisation and the team education module was completed (median 11 accesses/practice), the in-depth module was completed by ‘the healthcare professional responsible for asthma reviews’ (range 3-7 professionals/practice), and the asthma review template was successfully downloaded to the practice system. All six implementation practices received the baseline and first monthly audit and feedback report although there were delays in this process due to national-level governance changes. Practices’ perceptions of IMP^2^ART were encouraging. In general, they participated in the patient, professional and organisational implementation strategies and reported positive experiences of the trial.

**Conclusions:**

The study provides evidence that the IMP^2^ART trial is feasible, and the implementation strategy is acceptable with only minor adjustments to trial processes. The IMP^2^ART strategy is now being tested in a UK-wide cRCT [ref: ISRCTN15448074], evaluating implementation (action plan ownership) and health outcomes (unscheduled care).

## Introduction

An estimated 5.4 million people have asthma in the United Kingdom (UK) [1]. Each year asthma is responsible for over 6 million primary care consultations, 100,000 hospital admissions [2], and over 1000 deaths (20 a year in children under 14 years) [3]. Recent analysis suggests that lung conditions cost the UK economy £188 billion per year, and that developing and investing in self-management could reduce NHS costs by £128 million per year [4].

For decades [5], national and international guidelines have recommended that people with asthma should be provided with self-management education, reinforced by a personalised asthma action plan and supported by regular review with a healthcare professional [5–8]. Implementation of supported asthma self-management, however, remains poor in routine clinical care. For example, only half (52%) of respondents to a survey conducted by Asthma UK owned an asthma action plan [9] whilst, in our review of clinical records only 6% of people with asthma had a record of being provided with an action plan [10]. In 2014, the UK National Review of Asthma Deaths highlighted that only 23% of those who died from asthma were known to have been provided with an asthma action plan [11]. Further, according to the annual Asthma UK survey, almost 30% of respondents had not attended an annual asthma review [9].

In response to this, the IMP^2^ART (IMPlementing IMProved Asthma self-management as RouTine) programme has developed a facilitated implementation strategy, to embed supported asthma self-management in UK primary care. Based on multi-layer theoretical underpinning (a synthesis of integrated Promoting Action on Research Implementation in Health Services (iPARIHS) reflecting the pivotal role of facilitation in promoting implementation and the Capability, Opportunity, Motivation – Behaviour (COM-B) framework to promote individual behaviour change) the whole-system strategy operationalises a conceptual framework that addresses the needs of patients, the skills of professionals, and the organisational routines [12]. Facilitated by trained specialist asthma nurses, implementation of IMP^2^ART aims to promote patient-centred asthma reviews and increase ownership of asthma action plans, which will reduce unscheduled care. We will recruit 144 general practices, randomising them to either receive the IMP^2^ART implementation strategy or provide usual care [13].

This paper reports the internal pilot of the IMP^2^ART cRCT which builds on an extensive programme of implementation strategy development, iterative feasibility testing and refinement [14–17], and aimed to:

1. Observe the feasibility of the trial procedures (e.g., general practice recruitment and retention, initial data extraction, randomisation).
2. Explore engagement with the IMP^2^ART implementation strategy (e.g., access to implementation strategy components, acceptability to general practice teams) and fidelity with which the components of the implementation strategy were being adopted/adapted in the context of a trial.
3. Assess pre-specified trial progression criteria relating to the feasibility of trial procedures, engagement with the IMP^2^ART implementation strategy, and ability to recruit for the main trial.

## Methods

The reporting of this paper follows guidance for pilot implementation studies [18] and Standards for Reporting Implementation Studies (StaRI) [19].

### Design

We designed an internal pilot for the IMP^2^ART cRCT to assess the feasibility of and engagement with the IMP^2^ART trial. The trial is registered on The International Standard Randomized Controlled Trial Number database (ISRCTN15448074) and is sponsored by the Academic and Clinical Central Office for Research and Development (ACCORD), the University of Edinburgh and NHS Lothian Health Board. Ethical approval for the study was obtained from NHS Lothian (REC No: 19/EM/0279), NRS and HRA approval (NRS Ref NRS/19/256672), and local governance approvals as required for all sites. The participating practices signed contracts (see below under recruitment and randomisation); all individuals within the practices participating in interviews or other feedback provided written informed consent. Full details of the Hybrid II implementation cRCT (including the internal pilot) are described in the study protocol [13].

### Recruitment and randomisation

We aimed to recruit 12 general practices for the internal pilot (of the total 144 for the main trial) across England and Scotland. A sample size of 12 (approximately 8% of the main trial sample), was chosen as being both practical within our resources whilst allowing observation of the processes in sufficient practices representing diversity in our two key stratification variables (practice list size and socio-economic status) and to identify any changes that may be needed to the trial procedures/implementation strategy.

Recruitment re-commenced on 1^st^ January 2021 following a COVID-19 pandemic-related delay. General practices, some of whom had initially expressed interest pre-COVID, were recruited between 5^th^ October 2020 and 17^th^ March 2021. General practices were identified via local Clinical Research Networks, NHS Research Scotland, Optimum Patient Care, or via professional networks. As an implementation trial our inclusion/exclusion criteria were as broad as possible, but to be eligible, practices had to use one of the four electronic health record (EHR) systems commonly used in UK primary care (EMIS, SystmOne, Vision or Microtest) and agree to Optimum Patient Care (OPC: a social enterprise that leads quality improvement initiatives involving routine data extraction from practices https://optimumpatientcare.org) extracting anonymised routine coded data to measure the primary and secondary outcomes for the cRCT. General practices with a list size of >6000 patients were eligible to participate as, for statistical reasons, we wished to avoid cluster sizes <200. Approximately 6% of a UK general practice population have ‘active asthma’ defined as having a diagnostic code for asthma and having been prescribed asthma medication within the previous 12- months [20].

General practices who were interested in taking part in the trial met with a researcher, following which they were asked to sign a service agreement with OPC. We described this step as ‘recruitment’. OPC performed an initial routine data extraction to:

- Demonstrate that there were no insurmountable governance or technical problems,
- Establish that the general practice data would allow assessment of the primary health outcome (unscheduled asthma care),
- Confirm the data could be used in the baseline audit and feedback reports.

**Fig 1:**
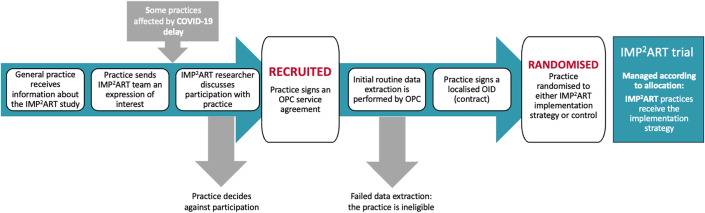
Recruitment and randomisation process. Abbreviations: OID = Organisational Information Document; OPC = Optimum Patient Care who extract the routine clinical data.

If the initial data extraction was unsuccessful, a practice would be ineligible to participate in the trial. Following a successful routine data extraction, the practice signed a localised Organisational Information Document (OID: the contract agreeing to participate in the cRCT) and was then randomised to either the IMP^2^ART implementation arm or usual asthma care (control) using predetermined stratifiers: practice list size, general practice training status of the practice and area-level socioeconomic conditions. Socioeconomic conditions were measured using the English and Scottish Indices of Multiple Deprivation 2019 (IMD and SIMD), which provide composite measures of social, economic, and environmental disadvantage for small areas (typically 1000–3000 residents). Deprivation and list size were dichotomised by the median. Randomisation was implemented in REDCap (Research Electronic Data Capture) software [21] by the programme manager (VH). Fig 1 displays the recruitment and randomisation process.

#### The IMP^2^ART implementation strategy.

Randomised general practices remain in the trial for 24 months. Full details of the implementation strategy and its evidence base are available in the trial protocol [13]. Briefly, general practices randomised to the implementation group received the whole-system implementation strategy directed at patients, professionals and the organisation, supported by expert nurse facilitation for 12 months. Components of the implementation strategy included:

- Patient resources (a patient-facing website that hosts a range of information about asthma (e.g., asthma triggers, asthma action plans, asthma reviews), waiting room posters, asthma review invitation letters) [14].
- Professional education (to raise awareness of supported asthma self-management and the importance of team working (an online team module (Module 1), and an individual module (Module 2) for those in the general practice most involved with delivering asthma care) [15].
- Organisational resources (an asthma review template embedded in the practice’s EHR system that guides the delivery of essential components of a patient-centred asthma review [16], and annual and monthly audit and feedback reports [17]).

The development and full details of these components can be found in their respective publications [14–17].

All implementation general practices received an initial IMP^2^ART workshop open to all members of staff (e.g., administrative staff, general practitioners (GPs), nurses, etc.) via Microsoft Teams, during which the IMP^2^ART facilitator discussed the baseline annual audit and feedback with the practice team and introduced components of the team module. The facilitator *(one of the four senior asthma nurses with facilitator expertise* *who were* *trained and supported to deliver the IMP*^2^*ART implementation strategy)* guided the practice to develop their own ‘team plan’ for implementing IMP^2^ART, discussed how core strategies could be adopted/adapted to suit the general practice clinical care routines, and identified additional strategies that might help individual practices. The workshop was scheduled approximately six weeks post-randomisation, during which several emails were sent to the practice providing implementation strategy resources (the patient resources, access to the team module, and the baseline annual and first monthly audit and feedback reports) in readiness for the workshop. Access to the individual module was provided following the workshop, during which details of staff (name, and email for the log-in, professional role) who wanted to complete the module were collected. Ideally, the asthma review template was uploaded to the practice EHR system prior to the workshop date.

General practices in the control arm of the trial continued with their usual asthma care and did not receive any of the components of the implementation strategy. However, they did receive standard versions of the extensive annual report covering all aspects of asthma care that OPC routinely provides to practices contracted to their quality improvement service. In contrast to the IMP^2^ART implementation arm annual reports, there was no focus on supported self-management, and the feedback on proportion of patients with an action plan is towards the end of the report.

#### Data collection and analysis.

The pilot evaluation used data collected from the start of recruitment until the initial implementation strategies had been delivered (approximately 4 months post-randomisation). This included data related to the feasibility of delivering the trial procedures (e.g., general practices recruited and retained, data extraction completed, randomisation, setting up the implementation strategy according to allocation, mailing of the OPC quality improvement questionnaire to a random sample of patients within a random sample of practices to measure asthma action plan ownership). To measure engagement with the IMP^2^ART implementation strategy in the context of trial participation we collected quantitative data related to: the workshops (e.g., delivery date, number of attendees, online evaluation form for staff), and the patient, professional, and organisational components of the implementation strategy (e.g., Google analytics to record number and duration of visits to the IMP^2^ART patient website, professional education completions and responses to the online evaluation form, asthma review template uploads, delivery of audit and feedback reports, facilitator contacts with general practices). To explore general practices’ responses to components of the implementation strategy and their experience of the trial, we conducted qualitative interviews with practice staff. In all data collection we offered flexibility in format and aimed for diversity in terms of roles within the team and experience of asthma care, monitoring for new themes to assess saturation. In addition, we interviewed the IMP^2^ART facilitators to explore their experience of facilitating the delivery of the implementation strategy (see S1 Appendix for the topic guides). Topic guides were developed with the multidisciplinary team (which included patients, clinicians, health psychologists, public health and health service qualitative research expertise) and were piloted with Patient and Public Involvement (PPI) colleagues (individuals with lived experience of asthma) prior to data collection. Quantitative data were analysed using descriptive statistics. We used a Framework Analysis [22] in NVivo 12 (QSR International, Massachusetts, US) of the qualitative data to answer the practical feasibility and acceptability questions for this internal pilot whilst remaining open to unexpected views.

#### Progression criteria.

Pre-specified progression criteria for the ‘stop/go’ decision were used to determine, in discussion with the Independent Steering Committee and the funder, whether to proceed from the internal pilot to the cRCT. The criteria (detailed in Table 1) reflected ability to recruit, evidence of general practice engagement with the IMP^2^ART implementation strategy, and patient engagement with the strategy. In parallel to the internal pilot, we report our progress with recruiting practices and ongoing preparations for randomisation in the main trial.

**Table 1:** Achievement against progression criteria for the ‘stop/go’ decision.

	Criterion	Threshold*		Achieved Description
Green	Amber
**1. Evidence of ability to recruit and randomise#**				
a	Successfully recruit and randomise 12 pilot general practices	12	9–11	**Achieved: 12 practices** We recruited and randomised 12 practices to the pilot trial (6 to the implementation group)
b	Confirmation that we could recruit general practices for the main trial (recruited ≈25% of the 132 additional practices required for the main trial)	30	10–24	**Achieved: 100 practices** A total of 100 practices (12 in the pilot + 88 for the main trial) had been recruited (i.e., signed the OPC agreement committing them to baseline data extraction). This substantially exceeded the threshold set for this criterion.
c	Local R&D approvals completed or on-going for recruited general practices	All relevant Scottish regions and English practices		**Achieved** We had NRS and HRA approvals for Scotland and England, and local R&D for eight Scottish regions and were waiting for approval from a further three. As participating practices were considered as ‘sites’, we required local R&D for individual practices in England.
**2. Evidence of general practice engagement with the IMP** ^**2**^ **ART implementation strategy (in the six pilot implementation practices)**				
a	Audit and feedback: Successful extraction of baseline data and preparation of feedback	5-6	3–4	**Achieved: 6 practices** All six implementation practices had a baseline data extraction and an annual audit report prepared. There was a delay with the first monthly reports due to changes in the data extraction algorithms required to meet new governance processes. This was subsequently resolved and all practices received one report confirming feasibility. Once the backlog clears this should not affect the trial going forward.
b	Facilitation: Initial IMP^2^ART practice workshop successfully undertaken and audit feedback reviewed	5-6	3–4	**Achieved: 6 practices** All six IMP^2^ART workshops were completed (via ‘MS Teams’). The median time between randomisation and the workshop was 12 weeks. Attendance ranged from 7 to 31 staff with representatives of medical, nursing and administrative staff; duration: 35 minutes to 1 hour and 45 minutes. Monthly feedback reports were discussed with all teams, but supporting practices to interpret their data was identified as an area for improvement.
c	Professional education: successful completion of the practice module, and a key professional undertaking the in-depth training module	≥4	3	**Achieved: 6 practices** All six practices accessed the team module (Module 1) with a median of 11 accesses/practice. Time spent on the module ranged from 10–58 minutes. Between 3 and 7 staff completed the in-depth training module (Module 2) from each of the six practices (Total: 29 professionals). Qualitative feedback suggested the educational modules were appreciated by staff with various levels of prior experience
d	Organisational engagement: adoption of one or more practical strategies (or if recently recruited plans to adopt)	≥4	3	**Achieved: 6 practices** All six implementation practices downloaded the template. There were some technological queries, and some practices asked about adapting details (e.g., how to add a field that was useful to their organisation). The qualitative feedback was generally positive. Other strategies commonly adopted were: using the audit report to target people who had not been followed up since a recent attack; using a monthly respiratory ‘huddle’ to review data and take action, targeting supported self-management interventions at patients with high levels of asthma risk. The monthly audit and feedback reports were under used in the first few months reflecting the delay with the first monthly reports (see 2a).
e	Evidence that the IMP^2^ART implementation strategy is acceptable and likely to be feasible	Qualitative data		**Achieved** We interviewed seven professionals/staff from the implementation practices, with only one of the practices not contributing an interview. Participants were generally very positive about the trial, saying that it will make *“a genuine difference for patient care”*. Specific feedback on facilitation, education modules, templates and patient resources suggested that the strategies were acceptable and uptake suggested that they were feasible to implement in routine clinical care.
**3. Evidence of patient engagement with the IMP** ^**2**^ **ART implementation strategy**				
a	Evidence that the IMP^2^ART implementation strategy is perceived to be engaging patients	Qualitative data		**Achieved** Several interviewees commented *that “It’s too early to tell about patient engagement at this stage”*, however, there were some indications about how the strategies may strengthen patient engagement with their asthma self-management, with some examples of positive experiences of using action plans in clinical practice.
b	Evidence from monthly audit reports of increasing provision of action plans in asthma reviews	≥4	3	**Too short a timescale to be clear** The timescale between the facilitation workshops (May/June) and the latest monthly report (July/August) was too short to judge the effect of the implementation strategy which was designed to have an effect over a year.
c	Evidence of activity on the IMP^2^ART patient-facing website	Observation of overall web-statistics		**Achieved** Usage of the patient-facing ‘Living with Asthma’ website since the first facilitation workshops was scheduled showed 444 unique page views. Interviewees were generally impressed by what was available; the most popular link was to the Asthma UK Action Plan. The facilitators planned to remind practices about the website during follow-up contacts, and several of the monthly ‘top-tips’ (included when delivering the audit and feedback) will include links to the website.

* ‘Green’ is the required threshold for progression; ‘Amber’ requires consideration of a recovery plan for achieving the ‘green’ threshold.

# This is a two-stage process. Recruitment refers to signing the OPC agreement committing to baseline data extraction. Practices with successful data extraction are eligible for the trial and are randomised (see Fig 1 and ‘Recruitment and Randomisation’ in the methods for details).

Abbreviations: HRA = Health Research Authority, NRS = NHS Research Scotland, OPC = Optimum Patient Care, R&D = Research and Development.

## Results

Recruitment and data collection for the pilot took place between October 2020 to September 2021. The results below integrate quantitative and qualitative findings exploring the feasibility of the trial procedures, engagement with the IMP^2^ART implementation strategy, and fidelity with which the components of the implementation strategy were adopted/adapted.

### Feasibility of recruitment and trial processes, and general practice characteristics

From a pool of 100 practices working through the recruitment/randomisation process (see Fig 1 and the Recruitment and Randomisation section), we randomised 12 general practices to participate in the IMP^2^ART pilot trial. Whilst we were mindful of the need for a diverse sample of practices, the successful conclusion of the recruitment/randomisation process was the key consideration.

All 12 practices were randomised to either the implementation arm (n = 6) or the control arm (n = 6). Seven of the 12 practices (implementation n = 3, control n = 4) were then randomly allocated to quality improvement questionnaire data collection. One practice withdrew post-randomisation to the control arm citing data sharing concerns and insufficient resources to support the questionnaire mailing (see Table 2).

**Table 2:** Practice characteristics and recruitment data.

General practice	Location and stratification characteristics				Recruitment		Randomisation	Allocation	
Trial ID	Clinical Research Network	List size^#^ Small or large	IMD^§^ More or less deprived	Training practice	Signed OPC agreement [T-x weeks]	Signed OID [T-x weeks]	Date [Time zero (T)]	Trial Arm	Quality Improvement Questionnaire
Practice 1	North West London	Small	Less deprived	Yes	−5	−1	30/03/2021	Implementation	No
Practice 2	Kent, Surrey and Sussex	Large	Less deprived	Yes	−23*	−1	30/03/2021	Implementation	No
Practice 3	Thames Valley and South Midlands	Large	Less deprived	Yes	−8	−1	30/03/2021	Implementation	No
Practice 4	Yorkshire And Humber	Large	More deprived	Yes	−3	−2	10/03/2021	Implementation	Yes
Practice 5	North West Coast	Large	More deprived	Yes	−14*	−2	10/03/2021	Implementation	Yes
Practice 6	Thames Valley and South Midlands	Large	Less deprived	Yes	−19*	−2	10/03/2021	Implementation	Yes
Practice 7	West of England	Small	Less deprived	Yes	−14*	0	10/03/2021	Control	Yes
Practice 8	Eastern	Large	Less deprived	Yes	−2	0	30/03/2021	Control	Yes
Practice 9	Eastern	Large	More deprived	Yes	−8	0	04/05/2021	Control	Yes
Practice 10	Yorkshire and Humber	Large	More deprived	Yes	−22*	−2	10/03/2021	Control	No
Practice 11	Kent, Surrey and Sussex	Large	Less deprived	Yes	−7	−2	10/03/2021	Control	No
Practice 12	Kent, Surrey and Sussex	Large	More deprived	Yes	−16*	−2	10/03/2021	Control	Yes

#Small or large practice compared to mean list size in England of 8,035.

§Deprived or affluent compared to median deprivation score (21.8) for England.

* Timeline delayed by COVID-19 pandemic.

Abbreviations: IMD = Index of Multiple Deprivation (England: https://www.gov.uk/government/collections/english-indices-of-deprivation); OPC = Optimum Patient Care (Data extraction: https://www.optimumpatientcare.org); OID = Organisational Information Document (research contract); T = Time zero (date of randomisation).

### Engagement with the IMP^2^ART implementation strategy

We conducted interviews (n = 7) with general practice staff (GP n = 3, nurse n = 3, practice manager n = 1) across five practices between July and September 2021. The interview topic guide was used flexibly to accommodate diversity in roles and the time available; the duration ranged from 11 to 39 minutes. In addition, we conducted interviews with three IMP^2^ART facilitators during August 2021 with interviews ranging from 41 minutes to 1 hour and 19 minutes.

#### IMP^2^ART workshops.

An IMP^2^ART workshop, led by one of the facilitators, was delivered successfully to all six general practices. Table 3 displays the timing of the workshop post-randomisation. On average, a workshop was delivered to a practice within 11.5 weeks of randomisation (range 7–14 weeks). The number of general practice staff who attended the workshops ranged from 7 to 31 per practice. GPs, nurses and administrative staff were represented in all practices; two practices included a pharmacist. The workshops ranged from 35 to 62 minutes in duration. Table 3 displays the number of attendees and workshop duration per practice.

**Table 3:** IMP^2^ART implementation strategy delivery and engagement.

General practice	Workshop		Patient resources	Professional education modules		Organisational strategies	
Trial ID	**Time from randomisation** [T+x weeks]	• number of attendees (n clinical)^†^ **•** duration*	**Information sent** [T+x weeks]	**Team module** • Completion rate, • Time spent, **•** Number of visits	**Individual module** • Number enrolled, • Number completed ≥80% (profession), **•** Time spent range^#^	**Template uploaded** [T+x weeks]	**A&F reports** Expected, Delivered
Practice 1	7	• 7 attendees (5 clinical) • 35 minutes	5	• 100% • 58 minutes • 6 visits to module	• 16 enrolled • 7 completed ≥80% (5 nurses, 1 pharmacist, 1 HCA) • 16–175 minutes	6	4 expected, 2 delivered
Practice 2	11	• 17 attendees (12 clinical) • 55 minutes	9	• 100% • 26 minutes • 1 visit to module	• 15 enrolled • 3 completed ≥80% (2 GPs, 1 HCA) • 35–78 minutes	12	3 expected, 2 delivered
Practice 3	12	• 13 attendees (12 clinical) • 50 minutes	10	• 100% • 45 minutes • 4 visits to module	• 11 enrolled • 4 completed ≥80% (2 GPs, 2 nurses) • 46–85 minutes	6	3 expected, 1 delivered
Practice 4	14	• 31 attendees (16 clinical) • 62 minutes	13	• 100% • 18 minutes • 49 visits to module	• 24 enrolled • 3 completed ≥80% (2 GPs, 1 pharmacist) • 15–80 minutes	10	3 expected, 2 delivered
Practice 5	11	• 25 attendees (10 clinical) • 56 minutes	9	• 100% • 10 minutes • 24 visits to module	• 17 enrolled • 8 completed ≥80% (2 GPs, 3 nurses, 1 student, 2 not stated) • 12–103 minutes	9	4 expected, 2 delivered
Practice 6	14	• 20 attendees (16 clinical) • 55 minutes	12	• 100% • 47 minutes • 15 visits to module	• 18 enrolled • 4 completed ≥80% (2 GPs, 2 nurses) • 27–115 minutes	11	3 expected, 3 delivered

†All clinicians from the practice were eligible for the individual educational module, including those unable to attend the workshop.

* The workshop duration commences after welcome and introductions when recording was commenced.

#Of those who completed ≥80%.

Abbreviations: A&F = Audit and Feedback reports, GP = General Practitioner, HCA = Health Care Assistant, T = Date of randomisation (see Table 2).

Across five practices, 17 general practice staff members filled out an online evaluation form following the workshops (in the sixth practice the staff did not have time to complete the evaluation form before rushing to their afternoon clinics). Respondees represented a range of roles within the practices (Advanced nurse practitioner 3, Pharmacist 1, Data Quality Assurance Manager 1, GP 5, Health Care Assistant 2, Patient Care Advisor, 1, Practice Nurse 1, Receptionist 3). 16 (94.1%) stated that they had accessed and viewed the team module prior to the workshop. 14 respondents (82.4%) either agreed or strongly agreed that the workshop improved their understanding of supported self-management for asthma and also helped them to understand their role in supported self-management of asthma. 16 (94.1%) either agreed or strongly agreed that the workshop content was organised and easy to follow. There was one suggestion for improvement to the workshop, which was to include a demonstration of the review template and other resources. When asked if there was anything staff would do differently having completed the workshop, six mentioned asthma action plans (e.g., discussing them with patients, printing them, or sending them to patients using the EHR), two staff mentioned becoming more knowledgeable or aware and up to date with processes in the general practice, two mentioned promoting attendance at asthma reviews, and two receptionist members of staff said they would ask patients more questions about their asthma care.

Results from the qualitative interviews found that general practice staff were positive about the workshops, as they provided a useful revision, and inspired healthcare professionals to make positive changes to the delivery of asthma care and to improve strategies in their practice.

*‘‘It [workshop] was good to have – to kind of reinvigorate my understanding of asthma basically…and to try and inspire my colleagues to engage with the IMP*^*2*^*ART module and also the templates.’’* (Practice 3, general practitioner)

One participant felt it was beneficial to their practice that all staff members were invited to the workshop, as it started conversations and prompted ideas of how organisational changes could be made to the provision of asthma care.

*‘‘I think it [workshop] was very important just because it gave everyone then a bit of understanding of what should be happening and how things should work....So it kind of got the whole staff talking about it, which was good.’’* (Practice 5, practice and research nurse)

#### Patient resources.

All six general practices were emailed the patient resources prior to the workshop. The email included a link to the IMP^2^ART ‘Living with Asthma’ website which included patient-facing pages, the waiting room posters, and suggested patient invitation letters [14]. Over the internal pilot period (when no main trial practices had been randomised), the patient-facing website received 444 unique page views, with an average of 1 minute and 8 seconds spent on each page.

The qualitative interviews found that the patient resources were mostly viewed as useful by general practice staff. For example, a number of participants valued the patient resources on the ‘Living with Asthma’ website.

*“I think being able to direct the patients to the website is really, really helpful as well so that they can go through that themselves and I’ve kind of gone through it and looked through what they will be accessing and what they can see. I think that’s really, really helpful for them”* (Practice 2, general practitioner)

However, some participants highlighted that there is already a breadth of online asthma information directed towards patients, and that they were likely to continue to signpost patients towards more familiar resources such as Asthma UK [now Asthma + Lung UK].

*‘‘Asthma UK’s is nice and easy to remember. I think, you know, in my head I’ve seen Asthma UK for so long as my reference option. So, I think I’m almost on kind of autopilot when I recommend it to patients really’’* (Practice 3, general practitioner)

There was a range of adoption of the IMP^2^ART waiting room posters. At the time of the pilot study, the UK was still under COVID-19 restrictions, and for this reason some practices either did not have posters in waiting areas, or rarely had patients in their waiting room. However, they were well received, and some practices suggested alternative strategies for displaying information.

*“We don’t because we haven’t got that many posters at the moment but we are thinking about putting it on our website”* (Practice 1, practice manager)

*‘‘We got all the IMP*^*2*^*ART stuff out, we’ve got posters in our waiting room, we’ve got an electronic board in the waiting room, on our Facebook page, on websites we’ve got that all rolled out’’* (Practice 4, general practitioner partner)

There were mixed findings in terms of the invitation letters. Some practices had adapted the IMP^2^ART developed invitation letters for use in their practice; others were either unaware of them or did not send invitation letters in their practice.

*“…in terms of the invitation letters and the messages. So we had some in place already but what I did is I took those and I sort of integrated them into our own ones as well. I thought the ones that were helpful in terms of just sort of providing things like almost kind of like the gold standard we’re aiming for so I’d need to have this level of information. So that was quite helpful to compare across”* (Practice 4, general practitioner partner)

#### Professional education modules.

**Team module (Module 1):** All six general practices were enrolled for the team module prior to their IMP^2^ART workshop. Each practice received one login for multiple use, which anyone in the practice team could access. All six practices completed the module (100%), spending between 10 minutes and 58 minutes over the course of between 1 and 49 visits.

A total of 21 members of staff across four practices completed an online evaluation form for the team module. 17 (81.0%) either agreed or strongly agreed that the module had improved their understanding of supported self-management in asthma. All agreed that the content of the course was organised and easy to follow. 17 (81.0%) either agreed or strongly agreed that the module helped them to understand their role in supported self-management of asthma. In response to an optional question asking staff if there is anything that they would do differently following viewing the team module, four mentioned they would identify high-risk patients, three said that they would encourage/highlight supported self-management more, two mentioned they would use the IMP^2^ART review template, two mentioned asthma reviews and processes to invite patients for these. Only two said there would be no change in their practice. Additionally, qualitative interview findings were generally positive.

*“Oh, it’s very useful, I learnt more… all this asthma that we have to be you know, mindful of these and all these things. But it’s just a reminder or refreshing my knowledge”* (Practice 1, practice manager)

**Individual module (Module 2):** In total, 101 general practice staff across the six practices asked to be enrolled for the team module. Of these, 29 completed at least 80% of the module (12 nurses, 10 GPs, 2 pharmacists, 2 health care assistants, 1 student, 2 not stated), with three or more clinical staff per practice completing the whole module (including some ‘additional resources’ sections). The average completion time was 66 minutes across an average of two visits (range 1–5). The minimum completion time was 12 minutes, though the participant had responded with appropriate text to all the questions. See Table 3 for further details for each practice. A total of 13 staff across five practices completed an online evaluation form after completing the individual module. All agreed that the module improved their understanding of supported asthma self-management, describing the content as organised and easy to follow, and applicable to their clinical practice. When asked what they would do differently having completed the module, answers included asthma action plans (e.g., co-create a personalised plan for every patient) (n = 8), motivational interviewing techniques (n = 4), and reviewing patients post attacks (n = 1).

There was very positive feedback about the individual module during the qualitative interviews.

*“I just love that education module two, it’s brilliant.”* (Practice 3, general practitioner)

It was considered that Module 2 increased awareness of supporting asthma self-management and refreshed clinician knowledge of asthma. One GP observed that it restored her confidence in asthma management which had become the expertise of the practice’s ‘skilled’ asthma nurses.

*‘‘It was quite interesting, especially for me because I’d only ever done training on asthma before through like websites and through Asthma UK… and what should be in the reviews because I was new to practice nursing… it helped my knowledge with asthma, and what should happen and about the treatment plans’’* (Practice 5, practice and research nurse)

*‘‘So, basically it taught and revised my knowledge of the asthma plan, and the importance of completing a plan... because it’s something that I haven’t done probably, in all honesty, since I was a GP trainee, which is about 10 or 12 years ago. So, that second module about where to find the plan, how to print the plan, how to complete the plan, has given me the confidence to print some out, which I now have in my little book in my desk, so I can just whip one out”* (Practice 3, general practitioner)

#### Organisational strategies.

**Audit and feedback reports:** All implementation group practices received both their annual baseline and first monthly report prior to their workshop, so these were discussed at the facilitated workshop. Control group practices received the standard annual report from OPC not customised to IMP^2^ART trial and with no facilitated discussion. Technical changes required to comply with new governance requirements resulted in delays in sending the monthly reports to all but one practice across the pilot period. Table 3 displays the number of expected monthly reports for the practice and the number of monthly reports that were actually delivered. The qualitative interviews found that most participants had not studied the audit and feedback reports.

*“I’ve not seen the latest one, no I haven’t, although I did pick it up briefly but I haven’t been through it yet. It’s on my to do list”* (Practice 4, general practitioner partner)

Participants who had viewed the reports were positive about them. One participant discussed the value of audit and feedback reports in helping to ensure that patients are appropriately reviewed following unscheduled care.

*‘‘If we miss the hospital letter, your report stated who is missing and you know that you haven’t been reviewed, and then that is very helpful for our clinician’’* (Practice 1, practice manager)

Healthcare professionals suggested that training on the audit and feedback reports would help with understanding how the data are generated and also the content of the reports.

*‘‘I think it would almost be helpful just to have a little bit more of an explanation in terms of where the numbers are pulled from… what codes or what did that researcher look for in the notes because even if they are actually being missed or is it that we’re not coding this properly… it’s about being quite helpful for clinicians understanding the report”* (Practice 4, general practitioner partner)

The IMP^2^ART facilitators also felt that further training on audit and feedback reports would be useful. One facilitator discussed a lack of confidence in the reports as a result of several issues including inaccuracies and delays.

*‘‘Maybe one of the areas that if I were doing it again with new people [both facilitators and practice staff] would be to think about how we can provide some sort of training and support to more quickly and better understand the audit and feedback reports and how to make best use of them’’* (IMP^2^ART facilitator 1)

**IMP**^**2**^**ART asthma review template:** Five out of the six practices had the IMP^2^ART asthma review template uploaded to the practice EHR system prior to their workshop. In the remaining practice, the template was uploaded eight days after the workshop. The qualitative interviews found that the asthma review template was well received by clinicians, it was seen as comprehensive and possibly superior to existing review templates.

*“It’s so comprehensive, it’s got everything that you might want”* (Practice 3, general practitioner

*‘‘So we’re actually using, obviously as we’re meant to,* [the IMP^2^ART] *template now. And I actually think it’s better than our original, it still ticks all the boxes, but I just think it’s quite straightforward and it’s quite, it’s relatively easy to use as well’’ (*Practice 5, practice nurse)

The IMP^2^ART facilitators also discussed the positive feedback they received from clinicians about the IMP^2^ART asthma review template describing it as *“really popular.”*

#### Facilitation.

Throughout the pilot period, facilitators logged 21 emails and 6 meetings (in addition to the workshops) across the six general practices. Practice staff discussed their IMP^2^ART facilitation experience in the qualitative interviews, and were positive about the role played by the IMP^2^ART facilitators.

*‘‘She [facilitator] was really excellent, she was very knowledgeable, very practical, she struck me as experienced and understanding what we do – you know, how life is in primary care’’* (Practice 3, general practitioner)

*‘‘Unless somebody’s facilitating, you sometimes you can kind of, like, not see the wood for the trees, or you don’t kind of consider the question in a slightly different way that they put it to you. So that then influences your management plans, or your action plans might be different because you hadn’t considered other perspectives when you’re making that plan’’* (Practice 5, advanced nurse practitioner)

There were some challenges around the facilitation, which had been switched to remote delivery because of COVID-19 restrictions. For example, IMP^2^ART facilitators had to learn to navigate the online meeting software (Microsoft Teams) as well as delivering the content of the workshops.

*‘‘Still very much a bit like a fish out of water and mainly because of the technology, because I’m used to doing an awful lot of face-to-face facilitating and training. And I’m not fantastically good with technology. And Microsoft Teams is not the technology that I’m fantastically familiar with. So I still struggle’’* (IMP^2^ART facilitator 2)

Time management also posed a challenge to facilitation of the IMP^2^ART strategies, with IMP^2^ART facilitators and practice staff struggling to find a suitable meeting time when all key staff members were able to attend (practices should receive the workshop and up to 10h of contact time with the facilitator across 12 months). Practices found it difficult to *“block out”* time during their working day to meet with facilitators; facilitators described “*a lot of to-ing and fro-ing to get people in the room”* as they tried to be flexible whilst working around their other commitments. Learnings from the pilot proved useful in streamlining the communication between IMP^2^ART facilitators and the pilot study practices.

### Trial progression criteria

The findings of this internal pilot demonstrated that we had met the progression criteria (see Table 1) and the main trial commenced randomisation in August 2021.

### Changes for the main trial

We paused randomisation for five months so that we could assess the learnings from the pilot and make any necessary changes before commencing randomisation for the main trial. No substantial changes were made to the implementation strategy or trial procedures, but some minor adjustments were made to the processes. Following the withdrawal of a control general practice, we defined a procedure in which the programme manager (VH) would be notified if a general practice was (or maybe) considering withdrawing so they could contact the practice promptly to address any concerns. In terms of the implementation strategy, we refined the set-up processes so that OPC met with the practice approximately two weeks prior to the workshop to upload the asthma review template and install the Practice and Patient Level Report Generator software tool (used to identify high-risk patients from the audit and feedback reports). We added SMS asthma review invitation templates to the patient resources, and also made very minor changes to a table in the monthly audit and feedback email to match the visuals of the attached report and added *‘Please feel free to circulate this email to members of staff in your practice’* to enhance engagement with the reports.

Finally, we made minor changes to the facilitation processes including reducing the number of slides used in the workshops so that facilitators have more time to highlight the resources available to the practice (e.g., the audit and feedback reports) and to discuss the practice’s team plan including identifying a champion in the practice to help drive progress. Remote workshops remained a necessity in the aftermath of the pandemic which had the advantage of improving access for part-time staff or in multi-centre practices. We provided support for the sessions not only to manage technical issues, but also to relieve the facilitator of administrative and/or research related tasks.

## Discussion

The IMP^2^ART internal pilot to a cluster randomised controlled trial assessed the feasibility of trial processes and engagement with the implementation strategy and demonstrated that we had met the progression criteria. We successfully recruited our target of 12 general practices to participate in the pilot, though the unique immediate post-pandemic context limits extrapolation to recruitment in a future trial.

The delivery of the implementation strategy to the six implementation practices was generally successful, and the strategy was typically well received. Minor adjustments were made to trial procedures with no substantial changes made to the implementation strategy. Previous feasibility studies had iteratively tested and refined the multiple components of the implementation strategy [14–17], then in a ‘pre-pilot’ we had combined the components in a unified strategy for delivery in four practices. At this stage we realised that using the team education module (Module 1) in the pre-pilot facilitation workshop was constraining the discussion. We therefore took a more ‘practice-centred’ approach, focussing the discussion on the individual practice interests/needs and capturing key priorities in a ‘practice plan’ which was followed up by the facilitator over the next 12-months [12] In addition, we had adapted the resources to a post-Covid context – including remote delivery of the workshop. The pilot trial was the first time these changes had been put into practice and the experience for the practices and the facilitators was a key focus for our pilot assessment.

A delay between recruitment (which required contracting with OPC for data extraction and their quality improvement service) and randomisation was intentional to ensure that we did not randomise a general practice that was unable to provide the routine data that provided the primary outcome (unscheduled care) as well as underpinning the audit and feedback strategy. The COVID-19 pandemic exacerbated this delay. Several of the practices had originally expressed interest early in 2020 before the pandemic forced us to postpone starting the trial. We re-established contact in Autumn 2020 when it seemed likely that we would be able to recommence in January 2021 but this added to the prolonged recruitment/randomisation timeline (see Fig 1).

Unfortunately, one general practice withdrew post-randomisation citing governance and resource issues with supporting delivery of the quality improvement questionnaire. The practice was allocated to the control arm of the trial, therefore withdrawal was not related to the demands of the implementation strategy, so does not raise concerns about the potential of IMP^2^ART to be scaled up in routine primary care in the UK (if successful).

There were delays in the delivery of the audit and feedback over the pilot period, with only one practice receiving the full complement of monthly audit and feedback reports. This was due to technical changes being required to comply with the latest NHS data-sharing opt-out over the summer of 2021 [23]. This specific issue has now been resolved, but supporting facilitators and practices to interpret their audit reports remains a challenge [17]. The 5-month follow-up reported in this paper captured the critical early trial interventions and allowed us to move on to recruiting for the main trial, but as an internal pilot the practices remained in the trial for two years and we continued to monitor their progress with follow-up and data extraction processes.

### Strengths and limitations

Minimal changes were needed due to the extensive development [24–29] and pre-piloting of the IMP^2^ART strategies and resources [14–17] which included input from patient and public involvement, a professional advisory group, current general practice staff and those currently living with and being treated for asthma in the UK. This broad context helped mitigate any lack of generalisability in practices recruited to the pilot trial and/or the views we were able to capture.

A limitation of the study was that no practices in Scotland were able to be recruited in time for the pilot (due to data extraction delays). However, the practices in the pilot were relatively diverse in terms of their geography in England, their list size, and deprivation status, which makes the findings more generalisable. All practices were teaching practices, reflecting the extensive involvement of primary care in training GP registrars, undergraduates, foundations doctors, nurses and other professionals but limiting applicability to non-training practices.

There are also some limitations with regard to the data on engagement with the implementation strategy that we were able to collect. For example, using Google Analytics we are unable to establish which specific practices engaged with the patient-facing website, but we rejected the option of practice-specific logins to avoid creating a ‘password barrier’ preventing easy use. Further, with regard to exploring the engagement with the professional education, Module 1 provided a team login (because it was designed as a team building resource), so we are unable to establish whether each visit was from a unique staff member – or indeed groups of staff members. Additionally, it should be noted that all clinical staff could request to be enrolled on the individual module (Module 2) – typically collected as a list during the workshop – even if they are not involved with asthma care delivery. This will have influenced the relatively low completion rates for the module though it was encouraging that at least one key clinician in all six implementation practices undertook the complete module.

Finally, from our pilot data we are restricted in the comments we can make on patient engagement, partly because of the short timescale, but also because the measures are either second-hand (views of practice staff on patient engagement) or imprecise (overall use of the website). Whilst this reflects the professional/practice target of the IMP^2^ART implementation strategies (and thus our outcomes) the main trial includes a ‘quality improvement questionnaire’ mailed to a random sample of patients from a random sample of practices which will allow some investigation of the impact on patients in participating practices [13,30].

## Conclusion

The internal pilot suggested that the IMP^2^ART programme was likely to be successful in recruitment and implementation delivery. The components of the implementation strategy are theoretically informed [12] and evidence-based (patient information [31], professional education [27], use of templates [29] and audit and feedback [32]) and our findings that these initiatives were generally acceptable and feasible in the context of supported self-management of asthma is encouraging. Additionally, general practice engagement in the pilot suggests that IMP^2^ART has the potential to be an effective strategy to improve the delivery of supported asthma self-management in UK primary care. Having met the progression criteria, the IMP^2^ART strategy is now being tested in a UK-wide cRCT, evaluating implementation (action plan ownership) and health outcomes (unscheduled care).

## Supporting information

S1 Appendix IMP^2^ART pilot interview topic guides. a) Healthcare Professionals and Administrators employed in IMP^2^ART implementation practices. b) Interview Topic Guide to be used with facilitators working with IMP^2^ART implementation practices.
(DOCX)
