# Supplementary material for: IMPlementing IMProved Asthma self-management as RouTine (IMP2ART) in UK primary care: An internal pilot for a cluster randomised controlled trial
Source: PLoS One. 2026 Mar 20;21(3):e0336745. doi: 10.1371/journal.pone.0336745 (PMC13004369; doi:10.1371/journal.pone.0336745)
Supplement: S1 Appendix — a) Healthcare Professionals and Administrators employed in IMP2ART implementation practices. b) Interview Topic Guide to be used with facilitators working with IMP2ART implementation practices. (DOCX) [file pone.0336745.s001.docx]

**S1. IMP^2^ART pilot interview topic guides**

1. **Healthcare Professionals and Administrators employed in IMP^2^ART implementation practices**

Thank you for agreeing to this interview. We would like to learn from you about your expectations and experiences of IMP^2^ART in the context of your role in asthma care in the practice.

Any information about you, and everything that you say will be kept strictly confidential. Your name and contact details will be kept securely at the University of Edinburgh and only used for the purpose of the study. Interviews will be audio recorded and everything that you tell us will be written out and anonymised before we review and analyse our findings.

*[Notes for researchers:*

- *If remote, could check in on where are you doing the interview? Shared space? Work? Home?*
- *Check in on how long they have got. If time is short prioritise questions in* ***green*** *and consider dropping those in* ***grey****.*
- *Feel free to re-order as you need to make the interview flow*

| **CONTEXT: your role in the practice, and your role in asthma care**  **Q: – Could we start by you giving me a bit of background on your role in the practice and in particular your role in asthma care**  Possible prompts   - Current role - Leadership role in practice/beyond? - Involvement/responsibility for asthma reviews? |
| --- |
| **INNOVATION: experience/practice delivering supported self-management in asthma before IMP^2^ART**  **Q: - Thinking about before you took part in IMP^2^ART can you tell me about how asthma care was delivered**  **Q: What barriers /opportunities to delivering supported self-management did you experience?**  Possible prompts   - Patient factors e.g. attendance at asthma clinics, engagement, multimorbidity - HCP factors e.g. access to/uptake of relevant training, skills, motivation - teamwork/ practice attitudes towards SSM - organisational issues   Probe - individual, practice |
| **RECIPIENTS: Views on IMP^2^ART strategies**  **Q: Can you describe the IMP^2^ART strategies that you used or are using in your practice – how helpful/unhelpful are these?**  **[prompt list.** *Note for researchers – we are not inviting suggestions for changing each individual resource or suggesting that you use this as a list* *to go through systematically.***]**   - **Professional resources** - Team education module - Individual education module - **Patient resources** - Action plan templates - On-line information resources (<http://livingwithasthma.org.uk>   - Links to information on asthma/asthma treatment   - Links to asthma-relevant COVID information   - New enhancing IMP^2^ART resources (Media Clips/infographics)   - New remote resources (Patient-facing aspects of tool kit) - Invitation letters - Waiting room posters - **Organisational strategies** - Review templates - Annual audit and feedback report - Monthly audit and feedback e-mails, top tips - Online ‘review’ questionnaire and workflow   **Views on how IMP^2^ART addresses (*or does not address*) barriers to delivering supported self-management.**  Possible prompts   - Patient factors e.g. attendance at asthma clinics, engagement, multimorbidity - HCP factors e.g. access to/uptake of relevant training, skills, motivation - teamwork/ practice attitudes towards SSM - organisational issues   ***** see facilitation*: IF THEY HAVE RECALL OR RESPONSE TO ANY OF THE STRATEGIES,**  **Q: When have you found it helpful to adapt, change or stop using** **IMP^2^ART strategies? How do you envisage using them in the future?** |
| **FACILITATION (WHAT AND HOW): Experience of adopting/adapting the IMP^2^ART implementation strategy**  **Q: Did you attend the IMP^2^ART workshop on Teams?**  Q: What was your experience of this?  Possible prompts:   - What did you think you/the practice gained from it? - What didn’t work or could have been improved? - How do you think will enable your practice /you to improve delivery of SSM with asthma patients? - Team plan – what recall from it, how helpful   *[reiterate we want to learn and find out what can be improved – confidential, won’t go back to the facilitators]*  **Q: What else have the facilitators done?** [*note for researchers – explore unprompted first, then could give examples of resources from page x that the facilitator may have shared or discussed]*  How has this supported you in delivering SSM?  **Q: How important do you think the facilitation was in changing your practice?** |
| **PATIENT ENGAGEMENT**  **Q: How do you feel patients are responding to the changes in self-management?**  Prompts:   - patient online resources (see list in facilitation): can you give any examples/ reports of usage or where/when you have recommended resources to patients? Reported patient experiences of accessing these resources? - Response to IMP^2^ART template invitation letters and SMS - Patient-centred consultation - in person and online – experience of template guided review, action plans. |
| **RESEARCH PROCESSES**  This interview is part of IMP^2^ART’s pilot phase, to help us learn how to hone the **research processes** for the rest of the trial.  We would **welcome comments** about any aspect of the research that was unclear or unduly difficult that could be improved.  We are particularly interested here in your **communication with the researchers** and **how we collect data from you** - e.g. the phone call/emails to determine eligibility for the trial, how you were prepared for the facilitation workshop, setting up interviews such as this.  Would you recommend participating in IMP^2^ART to another practice? What influenced your response?  We are also interested in learning more about **how IMP^2^ART workshop participants experienced the video-recording of the sessions**:   - How comfortable were you with having your contributions to the workshop recorded? Might you have contributed more and/or differently if the workshop had not been recorded? - Did you have any questions about the video recording of the workshops at the time or on reflection? To what extent did you feel you were able to give informed consent? - Would your feelings about being video-recorded on an online platform like MS Teams be different to being filmed at an in-person IMP^2^ART workshop? |
| **CLOSING**  **Is there anything else you’d like to add?**  Give your thanks for their time.  Next steps – e.g. describe briefly what you’ll do with their data, e.g. “We will send you a summary of our findings at the end of the study. Findings will be presented at conferences and published in a journal. We will be using all that we have learnt to develop a practical way to help doctors and nurses deliver supported self-management.” |

1. **Interview Topic Guide to be used with FACILITATORS working with IMP^2^ART implementation practices**

Thank you for agreeing to this interview. We would like to learn from you about your experiences of facilitating IMP^2^ART during the pilot in order to optimise support for facilitators in the full study

**CONTEXT: Role in IMP^2^ART**

Possible prompts

- What led you to become a facilitator in this study?
- Practices you have worked with so far

**Training and support**

Facilitator training

- what training did you do?
- views on its usefulness to prepare you for the role
- suggestions for the full study

Materials/information on practices provided before your first visit

- What was helpful?
- Suggestions for improving content, format and timeliness?

What else was helpful or would you have liked to support you in your role?

**Delivering IMP^2^ART**

Initial practice visits

- Can you talk us through 1-2 different practice visits.
- What did you feel worked well? What might you do differently in future?

IMP2ART strategies

- - Patients - Action plans, letters, website x
  - Staff – online training
  - Organisations - template, audit and feedback

When did you find it helpful to adapt, change or stop using IMP^2^ART strategies?

What advice would you give to IMP^2^ART facilitators in the full trial?

**Process evaluation methods: experiences, suggestions**

Observations of visits, training
